# Retracted: A Role for PPAR***γ*** in the Regulation of Cytokines in Immune Cells and Cancer

**DOI:** 10.1155/2015/982750

**Published:** 2015-02-02

**Authors:** PPAR Research

The paper titled “A Role for PPAR*γ* in the Regulation of Cytokines in Immune Cells and Cancer” [1], published in PPAR Research, has been retracted as it is found to contain a substantial amount of materials from published papers. The three most original source papers are (1) X. Y. Yang, L. H. Wang, K. Mihalic, et al., “Interleukin (IL)-4 indirectly suppresses IL-2 production by human T lymphocytes via peroxisome proliferator-activated receptor *γ* activated by macrophage-derived 12/15-lipoxygenase ligands,” Journal of Biological Chemistry, vol. 277, no. 6, pp. 3973–3978, 2002; (2) L. Széles, D. Töröcsik, and L. Nagy, “PPAR*γ* in immunity and inflammation: cell types and diseases,” Biochimica et Biophysica Acta, vol. 1771, no. 8, pp. 1014–1030, 2007; (3) L. H. Wang, X. Y. Yang, X. Zhang, et al., “Transcriptional inactivation of STAT3 by PPAR*γ* suppresses IL-6-responsive multiple myeloma cells,” Immunity, vol. 20, no. 2, pp. 205–218, 2004.
